# Seasonal Trace Element Contamination and Health Risk Assessment of the Mediterranean Limpet (*Patella caerulea*) from the Southern Black Sea

**DOI:** 10.3390/life16050806

**Published:** 2026-05-13

**Authors:** Oylum Gökkurt Baki

**Affiliations:** Department of Environmental Engineering, Sinop University, Sinop 57000, Türkiye; ogbaki@sinop.edu.tr; Tel.: +90-368-271-57-68

**Keywords:** biomonitoring, metalloid, marine pollution, non-carcinogenic risk, molluscs, Black Sea ecosystem

## Abstract

The Mediterranean limpet (*Patella caerulea*, Linnaeus, 1758) is a native species in Türkiye that is not yet a major commercial species but has potential for future commercialisation, particularly given the country’s substantial mollusc export market. This study aimed to evaluate seasonal and station-level variation in trace-element burdens in *P. caerulea* collected from the Sinop inner harbour (southern Black Sea coast, Türkiye) and to assess the associated trace-element–related non-carcinogenic health risks under a precautionary consumption scenario. Spatial and seasonal variations in the concentrations of 10 trace elements (Mn, Fe, Co, Ni, Cu, Zn, Cd, Pb, Hg, and total As) were analysed in specimens collected seasonally from autumn 2022 to summer 2023. Permutational multivariate analysis of variance revealed that season was the primary factor influencing trace element concentration variability, accounting for 76.9% of the total variance, followed by station (11.2%) and the season × station interaction (7.2%). All elements varied significantly among seasons (Kruskal–Wallis, *p* < 0.001), with maxima in autumn and minima in winter. Spatial differences were significant only for Mn, Co, Pb, Zn, and Hg, indicating localised sources. A human health risk assessment was performed for 6-year-old children, 12-year-old children, and adults. Total target hazard quotient (TTHQ) values were <1 across all groups; however, Cd was the dominant contributor, with the highest value observed in children (max TTHQ = 0.94). TTHQ followed the seasonal contamination pattern, peaking in autumn. Even under the high-consumption scenario, TTHQ for *P. caerulea* from the Sinop inner harbour remained below the non-carcinogenic risk threshold. The strong seasonal signal supports its use in locally focused biomonitoring, while the health-risk assessment should be limited to the analysed trace elements and associated non-carcinogenic effects.

## 1. Introduction

The Mediterranean limpet, *Patella caerulea* (Linnaeus, 1758), locally known as ‘Çin şapkası’ (“Chinese hat”), is a herbivorous gastropod widely distributed across the Mediterranean Sea, adjacent Atlantic islands, and Türkiye’s Black Sea coast. This broad distribution makes it well-suited for regional biomonitoring [1,2,3,4]. Biomonitoring programmes frequently rely on sessile, widespread organisms to assess localised contamination [5,6,7,8]. Accordingly, *P. caerulea* has been extensively validated as an effective bioindicator of marine pollution throughout the Mediterranean due to its sedentary lifestyle, abundance, and high capacity to bioaccumulate contaminants [4,9].

As biomonitors integrate local contamination over time, the trace-element burdens measured in their tissues are directly relevant to seafood consumption–associated human health risks. Several of the trace elements analysed have recognised or proposed physiological roles at low concentrations, although excessive exposure may still be harmful. In contrast, Cd, Pb, Hg, and total As are of primary toxicological concern [10,11,12].

Beyond its ecological role, *P. caerulea* also intersects with human health and culture. Historically regarded as a ‘famine food,” limpets provided a vital protein source for coastal communities during periods of scarcity [13]. Today, this perception has shifted; traditional seafood is increasingly valued as part of tourism’s authentic culinary heritage, where consuming local dishes is linked to cultural engagement and memorable travel experiences [14].

These food safety implications are highly relevant in Türkiye, which harvested ~26.5 thousand tons of marine molluscs in 2024 [15], the majority of which were exported to the EU, South America, the United States, Japan, and China [16,17,18]. Although *P. caerulea* is not yet a major commercial species and remains primarily consumed by local communities [19], its potential for future commercial exploitation is evident, given Türkiye’s robust mollusc export market [16]. Assessing contamination levels in this species is therefore important both for environmental monitoring and for screening trace-element-related non-carcinogenic risks associated with consumption.

Although *P. caerulea* has been widely studied as a biomonitor in Mediterranean and Aegean coastal systems [4,20,21,22,23,24,25,26,27], information from the low-salinity southern Black Sea remains limited. This study addresses this regional gap by evaluating the seasonal and small-scale spatial variability of trace elements in *P. caerulea* from a semi-enclosed harbour environment on the Sinop coast. In doing so, it provides ecosystem-specific evidence on the use of this sedentary gastropod as a local biomonitor under Black Sea conditions and complements this ecotoxicological perspective with age-stratified screening of trace-element-related non-carcinogenic exposure.

Accordingly, this study investigated the spatial and seasonal dynamics of ten trace elements in *P. caerulea* from the Sinop inner harbour on the southern Black Sea coast. Specifically, the study: (1) quantified trace-element concentrations across four seasons and three stations; (2) used multivariate analyses to determine the effects of season, station, and their interaction on the overall trace-element profile; and (3) assessed trace-element-related non-carcinogenic health risks for children and adults using the target hazard quotient (THQ) and total THQ (TTHQ) under a precautionary high-consumption scenario. This health-risk component should be interpreted as a screening-level assessment limited to the trace elements analysed, rather than as a comprehensive seafood safety evaluation.

## 2. Materials and Methods

### 2.1. Study Area

**Figure 1:**
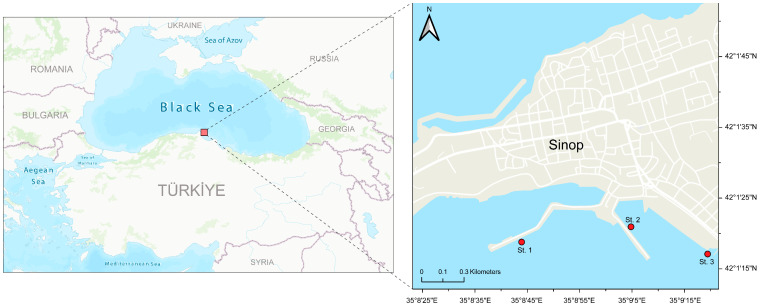
Map of the sampling stations in Sinop inner harbour, southern Black Sea, Türkiye.

This study was conducted along the southern coast of the Black Sea in Sinop, Türkiye (Figure 1). Three sampling stations (St. 1, St. 2, and St. 3) were selected within the Sinop inner harbour to represent distinct micro-environments and to evaluate local gradients in a semi-enclosed system. This area is characterised by significant anthropogenic pressures, including harbour operations, industrial discharges and urban wastewater inputs, which are compounded by limited water circulation. The local climate features average temperatures of 23.16 °C during summer (June–September) and 10.15 °C in winter (December–March). Although this design enables repeated seasonal sampling under comparable hydrodynamic conditions and characterises spatial heterogeneity within the harbour, it is not intended to represent the entire Sinop coastline or the wider Black Sea.

### 2.2. Sample Collection and Preparation

To assess temporal variation, *P. caerulea* specimens were collected seasonally (autumn 2022 to summer 2023) from rocky shorelines at three stations. In total, 180 individuals were collected in the field (15 individuals per station per season). Specimens were detached from the substrate using a plastic knife, placed in sterile polyethylene bags, and transported to the laboratory under refrigerated conditions.

In the laboratory, adherent material (i.e., sand and algal debris) was removed, shell length and wet weight were recorded, and soft tissues were excised using sterilised tools. To minimise potential size-related effects on trace-element concentrations while maintaining balanced representation across space and time, a size-matched subset was selected for chemical analysis. Specifically, 84 individuals were included (*n* = 7 individuals per station × season; 3 stations × 4 seasons), selected within each station × season group based on shell length and wet weight comparability. Where more than seven individuals met the size-matching criteria, individuals were selected at random from the eligible set. Soft tissues from the seven individuals within each station × season group were then pooled and homogenised to obtain one composite sample per group (12 composites in total), which served as the biological unit for subsequent chemical analysis. Excised tissues were stored at −20 °C until analysis and transferred to the analytical laboratory with the cold chain maintained.

Mean (±95% CI) shell length of the analysed specimens ranged from 4.11 ± 0.04 cm (winter) to 4.46 ± 0.02 cm (autumn), while mean wet weight ranged from 10.30 ± 0.38 g to 12.96 ± 0.35 g across seasons. Age was not determined; therefore, shell length and wet weight were used as size descriptors of the analysed individuals.

### 2.3. Trace Element Analysis (ICP-MS) and Quality Assurance

Soft tissues separated from the shells were homogenised in the laboratory using clean procedures designed to minimise contamination and were weighed in pre-weighed glass containers. The homogenised tissue samples were oven-dried at 105 ± 2 °C for 24 h. Repeated weighing was performed to verify mass stabilisation after drying, and the dried material was re-homogenised before subsampling for digestion. For each station × season composite, three independent aliquots of the dried homogenised tissue were digested and analysed separately, yielding three analytical determinations per composite and 36 determinations in total. Trace-element concentrations were determined on a dry-weight basis and are reported as mg kg^−1^ dw. For the human health risk assessment, dry-weight concentrations were subsequently converted to wet-weight concentrations as described in Section 2.4.

For digestion, approximately 0.5 g of dried homogenised tissue was accurately weighed into Teflon microwave digestion vessels and treated with 7 mL suprapur-grade HNO_3_ 65% and 1 mL H_2_O_2_ 30% (7:1, *v*/*v*). Closed-vessel microwave digestion was performed using a biological-tissue digestion programme. The temperature was increased to 200 °C over 15 min and then held at 200 °C for a further 15 min. After cooling, the digests were quantitatively transferred to 50 mL polypropylene tubes and diluted to a final volume of 50 mL with ultrapure water.

Concentrations of Mn, Fe, Co, Ni, Cu, Zn, Cd, Pb, Hg, and total As were determined using an Agilent 7700X (Santa Clara, CA, USA) inductively coupled plasma–mass spectrometer (ICP-MS). Instrumental response and signal drift were monitored and corrected using an Agilent internal standard solution introduced continuously during sample analysis.

External calibration was performed using certified Agilent multi-element and Hg standard solutions. Calibration standards were prepared in an acidified ultrapure-water matrix containing 1% HNO_3_ and 0.15% HCl. The calibration blank consisted of the same acid matrix without analytes. Calibration levels were 0, 0.5, 1, 5, 10, 50, and 100 µg L^−1^ for the multi-element calibration and 0, 0.1, 0.2, 1, 2, 10, and 20 µg L^−1^ for Hg.

Quality assurance and quality control were maintained through procedural blanks, triplicate analytical determinations, internal-standard correction, and certified reference material analysis. Accuracy was assessed using certified reference material TORT-2 lobster tissue, which was processed within the same analytical workflow as the samples. Analytical precision was within ±10%, and element-specific recoveries for the certified target analytes ranged from 90% to 100%. Because Hg was included among the target analytes and may be sensitive to sample-preparation conditions, the acceptability of Hg determination was evaluated through the same CRM-based quality-control procedure.

### 2.4. Assessment of Human Health Risk

Potential non-carcinogenic health risks associated with *P. caerulea* consumption were evaluated using the THQ for individual elements and the TTHQ for combined exposure. Risk was assessed for three consumer groups representing different body weights: children aged 6 years (22 kg), children aged 12 years (44 kg), and adults (72 kg).

THQ and TTHQ were calculated using the following equations:

(1) $$THQ=\frac{C_{w}\times EF\times ED\times FIR}{BW\times AT\times RfD_{o}}\times 0.001$$

(2) $$TTHQ=\sum_{i=1}^{n}TH\;Q_{i}$$

In Equation (1), *C*_w_ is the element concentration in edible tissue on a wet-weight basis (mg kg^−1^ wet weight), *EF* is the exposure frequency (365 days year^−1^), *ED* is the exposure duration (70 years), *FIR* is the food ingestion rate (7.4 g person^−1^ day^−1^), *BW* is the body weight (kg) of the relevant consumer group (22, 44, and 72 kg, as above), *AT* is the averaging time for non-carcinogenic effects (*AT* = *ED* × 365 days), and *RfD*_o_ is the oral reference dose (mg kg^−1^ day^−1^). A factor of 0.001 converts the ingestion rate from grams to kilogrammes.

The oral reference dose values (*RfD*_o_) used in this study were 0.14 (Mn), 0.7 (Fe), 0.0003 (Co), 0.02 (Ni), 0.04 (Cu), 0.3 (Zn), 0.001 (Cd), 0.0001 (Hg), 0.0003 (As) [28] and 0.0035 (Pb) [29]. Within the present framework, the Pb value was retained as part of the same screening-level THQ approach applied to the other trace elements; accordingly, Pb-related THQ results should be interpreted as conservative screening indicators within the broader limitations of threshold-based risk assessment.

A precautionary ingestion rate was applied using the global average mollusc consumption rate (7.4 g person^−1^ day^−1^) [30] rather than the lower national average reported previously (1.01 g person^−1^ day^−1^) [31], to provide a conservative and internationally comparable risk assessment. Because age-specific consumption data for *P. caerulea* or comparable local mollusc intake were not available, the same ingestion rate was applied to all consumer groups as a screening-level conservative assumption. This approach was not intended to imply identical real-world consumption by adults and children; rather, it was used to provide an upper-bound exposure scenario while avoiding the underestimation of potential risk in children. Age-related differences in exposure were still accounted for through body weight.

Element concentrations measured on a dry weight basis (mg kg^−1^ dw) were converted to wet weight (mg kg^−1^ ww) using a moisture content of 79.8 ± 0.8% [32]. For total arsenic (As), only 1% of the measured concentration was used in the THQ calculation, as the more toxic inorganic form typically constitutes ~1% of total arsenic in seafood [33,34,35].

THQ values (Equation (1)) were calculated using C_w_ concentrations from each analytical determination (after dry-to-wet conversion), and TTHQ (Equation (2)) was obtained by summing THQ values across elements for each determination. Results were summarised by station and season for each consumer group (mean ± 95% CI).

### 2.5. Statistical Analysis

The Shapiro–Wilk test was used to assess the assumption of normality for all element concentration data. Since the data were not normally distributed, nonparametric tests were applied. The Kruskal–Wallis *H*-test was used to identify significant differences in element concentrations among stations and seasons. When significant differences were found, Dunn’s post hoc test with Bonferroni correction was used for pairwise comparisons.

Multivariate analyses were conducted on log(x + 1)-transformed concentration data derived from the 36 analytical determinations (12 station × season composites × 3 aliquot determinations) to assess the combined variation in the ten trace elements and to evaluate the relative influence of spatial and temporal factors. Permutational multivariate analysis of variance (PERMANOVA) was performed on a Bray–Curtis dissimilarity matrix with 999 permutations using the adonis2 function in the vegan package [36]. A sequential (Type I) model (element_data ~ season + station + season:station) was used to partition the variance explained by season, station, and their interaction.

Principal Component Analysis (PCA) was conducted to visualise the multivariate structure of the data. Additionally, hierarchical cluster analysis using the group-average linkage method on the Bray–Curtis similarity matrix was performed to generate a dendrogram illustrating sample similarities. Spearman’s rank-order correlation analysis (*r*_s_) was also used to explore the relationships among the measured trace elements, and the results were visualised in a correlation matrix. In line with the study design, these correlations were treated as descriptive patterns of co-variation rather than mechanistic evidence of common sources or uptake pathways.

All statistical analyses were conducted using R (version 4.5.2), with a significance level set at *p* < 0.05.

## 3. Results

Trace-element concentrations in *P. caerulea* collected from three stations within the Sinop inner harbour showed clear temporal and fine-scale spatial structuring. At the within-harbour spatial scale investigated, multivariate partitioning based on the 36 analytical determinations indicated that season explained the largest proportion of the overall trace-element variation (PERMANOVA; Table 1), accounting for approximately 77% of the multivariate variance. Station contributed a smaller but significant fraction, approximately 11%, indicating a secondary spatial signal within the harbour. The season × station interaction accounted for a modest proportion, approximately 7%, suggesting that the seasonal pattern was broadly consistent, although the magnitude of seasonal differences varied among stations. The remaining unexplained variation, approximately 5%, likely reflects within-composite analytical variability and microscale environmental variability not explicitly captured by the sampled factors. Overall, the PERMANOVA results indicate that temporal variation was the dominant structuring factor, with station-level variation representing a weaker but detectable within-harbour effect.

### 3.1. Seasonal Variation in the Concentrations of Elements

**Figure 2:**
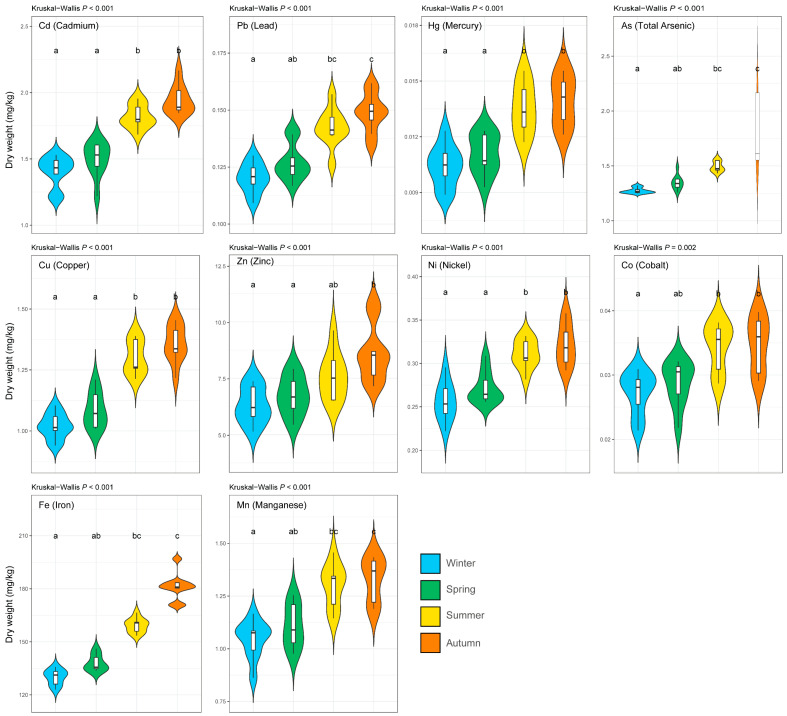
Seasonal variation in trace-element concentrations in the Mediterranean limpet (*Patella caerulea*). Concentrations are expressed as mg kg^−1^ dry weight. Different lowercase letters indicate significant differences among seasons (*p* < 0.05).

Element concentrations differed significantly among seasons for all 10 trace elements analysed (Kruskal–Wallis, *p* < 0.001 for each element; Figure 2). A consistent seasonal pattern was evident across both essential and toxic elements: concentrations were generally highest in autumn and lowest in winter, with intermediate values observed in spring and summer. This concordant directionality across the full element suite indicates that temporal variation exerted stronger control on accumulation than within-harbour spatial differences. The seasonal response was therefore broad and consistent across the analysed elements, rather than being restricted to isolated element-specific changes.

### 3.2. Spatial Variation in the Concentrations of Elements

**Figure 3:**
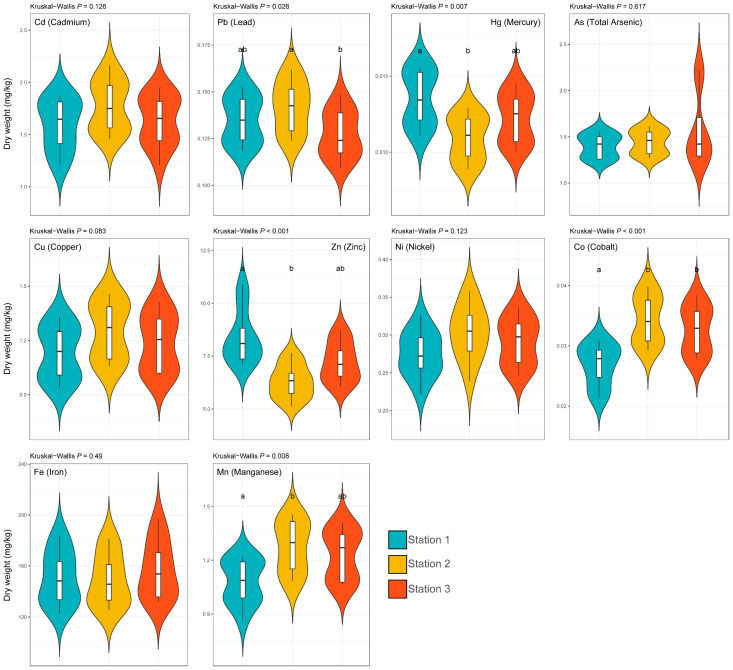
Spatial variation in trace-element concentrations in the Mediterranean limpet (*Patella caerulea*) among the three sampling stations. Concentrations are expressed as mg kg^−1^ dry weight. Different lowercase letters indicate significant differences among stations (*p* < 0.05).

Spatial differences among stations were significant for Mn, Co, Pb, Zn, and Hg (Kruskal–Wallis, *p* < 0.05; Figure 3). In contrast, Fe, Cu, Cd, Ni, and total As did not show significant station-level differences (*p* > 0.05), suggesting that these elements were more uniformly distributed across the three stations or that seasonal forcing outweighed local spatial gradients for these analytes. These results indicate that spatial heterogeneity within the harbour was element-specific and less consistent than the seasonal pattern.

### 3.3. Multivariate Patterns and Association of Elements

#### 3.3.1. Cluster Analysis

**Figure 4:**
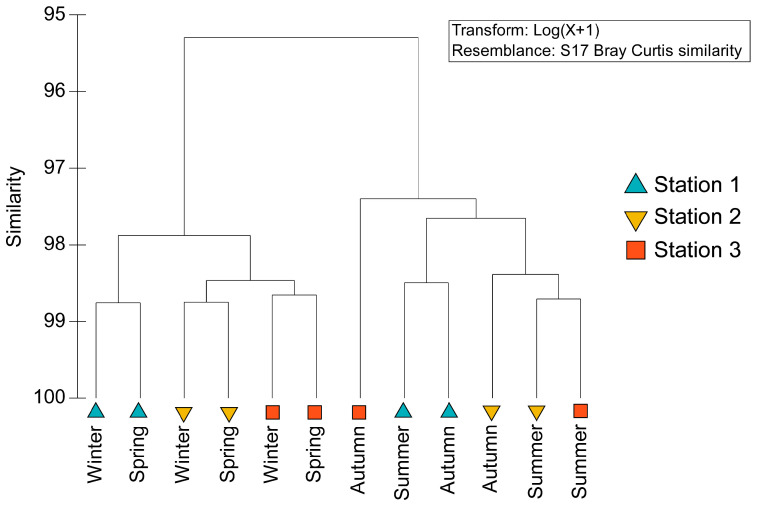
Hierarchical clustering of the Mediterranean limpet (*Patella caerulea*) samples based on trace-element concentration profiles using Bray–Curtis similarity.

Hierarchical clustering based on Bray–Curtis similarity separated samples primarily by season (Figure 4). Winter and spring formed a high-similarity group (>98%), whereas autumn and summer formed a second cluster with lower within-group similarity, approximately 97.5%. Autumn and summer were further separated as distinct sub-clusters within this second group. The intermixing of stations within the seasonal clusters indicates that multielement profiles were structured more strongly by season than by station.

#### 3.3.2. Principal Component Analysis

**Figure 5:**
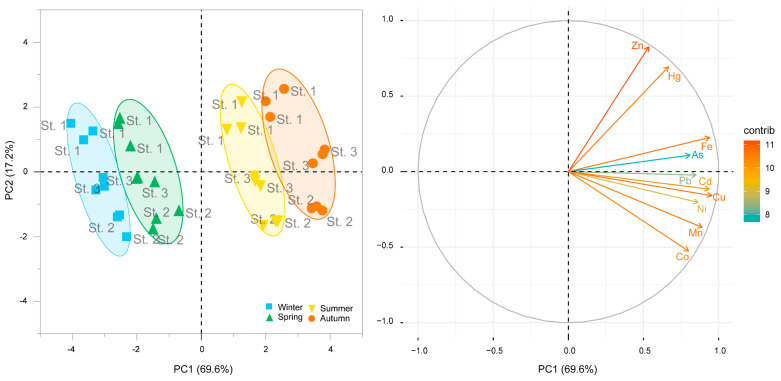
Principal Component Analysis (PCA) scores plot of trace element concentrations in the Mediterranean limpet (*Patella caerulea*). Samples are grouped by season (ellipses and colours) and labelled by station (St. 1, St. 2, St. 3).

Principal component analysis provided a complementary visual summary of the multivariate structure (Figure 5). The first two axes explained 86.8% of the total variance, with PC1 accounting for 69.6% and PC2 for 17.2%. Samples were separated mainly along PC1, with winter and spring positioned toward negative scores and autumn and summer toward positive scores. This ordination pattern is consistent with the PERMANOVA and clustering results, indicating a clear temporal structure in the multielement dataset.

#### 3.3.3. Spearman Correlation

**Figure 6:**
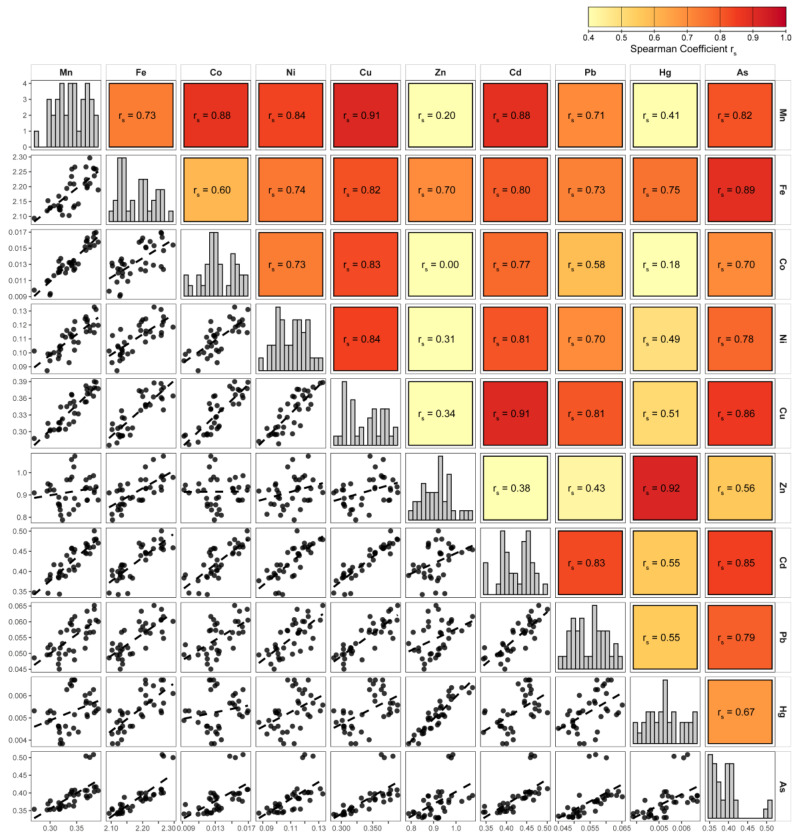
Spearman correlation matrix for the ten trace elements analysed in the Mediterranean limpet (*Patella caerulea*). The upper panel displays Spearman’s rank correlation coefficient (*r*_s_). The lower panel shows the corresponding scatterplots for each pair of elements, and the diagonal displays the distribution histogram for each element.

Most trace elements were positively correlated, indicating broad covariation in tissue concentrations (Figure 6). Strong associations were observed between Cu and Mn (*r*_s_ = 0.91), Cu and Cd (*r*_s_ = 0.91), and Fe and As (*r*_s_ = 0.89). Zn and Hg formed a distinct pair, exhibiting a strong mutual correlation (*r*_s_ = 0.92) and comparatively weaker correlations with several other elements. These correlations describe patterns of covariation among elements and should be interpreted descriptively rather than as evidence of shared sources or uptake pathways.

### 3.4. Assessment of Human Health Risk

Across all seasons, stations, and age groups, both THQ and TTHQ remained below 1 across the analytical determinations, indicating no exceedance of the non-carcinogenic benchmark under the assessed consumption scenario (Supplementary Table S1). TTHQ showed a clear seasonal increase from winter to autumn across all stations and age groups, broadly mirroring the seasonal pattern observed in trace-element concentrations.

For 6-year-old children, TTHQ increased from approximately 0.64–0.69 in winter to 0.87–0.94 in autumn, with the maximum value observed at St. 2 in autumn (TTHQ = 0.9392). For 12-year-old children, TTHQ rose from approximately 0.32–0.34 in winter to 0.43–0.47 in autumn. For adults, TTHQ increased from approximately 0.19–0.21 in winter to 0.27–0.29 in autumn.

Cadmium was the dominant contributor to cumulative risk in all age groups. As expected from body-weight scaling in the exposure calculation, risk estimates were consistently higher in children than in adults (Supplementary Table S1). Although all values remained below the non-carcinogenic threshold, the highest autumn estimates for young children were closest to 1, indicating that seasonal variability is relevant for interpreting exposure estimates.

## 4. Discussion

Coastal marine ecosystems, particularly semi-enclosed basins such as the Black Sea, face increasing pressure from anthropogenic activities, including industrial discharges and urbanisation, which introduce contaminants into the marine environment [37,38,39,40,41,42]. Both essential and non-essential (toxic) trace elements merit attention because they persist, can bioaccumulate through food webs, and may pose risks to consumers when exposure is elevated or prolonged [10,11,12]. In semi-enclosed harbour systems, restricted water exchange can enhance the retention of suspended material and intensify small-scale exposure heterogeneity, strengthening the value of sedentary molluscs as locally integrated biomonitors. This ecological relevance is mirrored by the public health dimension, because tissue burdens provide the first screening of exposure potential for locally harvested seafood [43,44].

### 4.1. Dominant Effect of Seasonality

PERMANOVA identified season as the dominant source of variation in trace-element profiles, explaining 76.9% of the total variance. Similar seasonal control has been widely reported in biomonitoring studies, where temporal dynamics shape uptake and depuration, dietary exposure, and organismal physiology [8,27,45,46]. More broadly, regional aquatic monitoring has also shown that environmental variables, nutrient and metal/metalloid concentrations, water-quality indices, and biological assemblages may vary more clearly through time than among sampling locations, reinforcing the importance of seasonal sampling in contamination assessment [47]. All 10 elements peaked in autumn and were lowest in winter. *P. caerulea* has been described to have comparable warm-season enrichment in other settings and has often been linked to higher metabolic activity and feeding during warmer periods [4,46]. The shared seasonal direction across essential and toxic elements implies a broad seasonal forcing rather than isolated element-specific events.

Autumn maxima may reflect cumulative exposure integrated over the preceding months rather than only sampling conditions. Summer can coincide with intensified shoreline use and harbour activity in an urbanised inner harbour, while the transition to autumn may increase contaminant delivery through early rainfall/runoff pulses, resuspension of particle-bound metals and seasonal shifts in suspended matter dynamics. Similar interpretations have been proposed for Turkish coastal systems, where seasonal variability in metal burdens has been associated with terrestrial inputs and hydrodynamic confinement, and where suspended matter composition in Black Sea ports varied across both space and season [39,48]. However, seasonal patterns are not universal. In the İskenderun Gulf (Mediterranean Türkiye), *P. caerulea* showed winter maxima for several elements (Cu, Zn, Fe, Ni, and Co), which is in contrast to the pattern observed here [27]. Mixed seasonal behaviour has also been reported elsewhere; for example, depending on local sources and pathways, Cu and Zn may peak in summer while Cd and Pb peak in autumn [49]. Taken together, these studies indicate that seasonality is a primary driver, but the direction and magnitude of seasonal change depend on temperature regime, food availability, terrestrial inputs, and the organism’s biological state. Because age, reproductive condition, growth rate, and concurrent environmental covariates were not quantified here, mechanistic interpretation remains plausible rather than demonstrative and should be treated accordingly.

### 4.2. Spatial Variation and Localised Contamination

Station explained a smaller but significant fraction of variance (11.2%), with significant differences for Mn, Co, Pb, Zn, and Hg. Spatial heterogeneity of this type is consistent with previous biomonitoring studies of *P. caerulea*, which often detect site-specific differences driven by local inputs and microhabitat features [23,27,45,49]. Such heterogeneity is ecologically meaningful within semi-enclosed harbours because a sedentary grazer can register fine-scale contrasts in exposure.

Elevated Zn and Hg concentrations at St. 1 and higher concentrations of Mn, Co, and Pb at St. 2 suggest that localised sources or pathways differ among harbour micro-environments (Figure 3). Comparable station-level contrasts have been linked to industrial or urban discharge proximity and to gradients between impacted and less impacted sites [27,49]. In this setting, differences in runoff influence, shoreline use, vessel-related activity, and the deposition or retention of particle-bound contaminants may be reflected. Turkish studies have also shown strong spatial variability among nearby ports and coastal stations in both metal contamination and suspended matter characteristics [39,40,44].

The significant season × station interaction (7.2%) indicates that spatial contrasts are not temporally constant (Table 1). Seasonal shifts in station “ranking” have been observed in other Turkish coastal studies [45], supporting the view that local inputs and hydrodynamics fluctuate throughout the year. Stations were selected to represent distinct harbour micro-environments with comparable shoreline type and depth and year-round accessibility for repeated sampling; therefore, inference is limited to the inner harbour and should not be extrapolated to the entire Sinop coastline or the wider southern Black Sea.

### 4.3. Comparison of Trace-Element Concentrations

A literature comparison suggests moderate contamination in the Sinop inner harbour within the context of available *P. caerulea* studies (Table 2). Cd concentrations exceeded the historical values reported for Sinop in the early 1990s [50], while remaining below the ranges reported for some more impacted Mediterranean localities [22,23]. Zn showed a similar pattern, remaining lower than the values reported from the Pontine Islands [23]. These comparisons imply that the detected contamination pressure did not reach the upper ranges reported for heavily impacted sites in the studies used for reference. Comparisons across studies should be interpreted cautiously because year, season, site characteristics, tissue preparation, analytical methods, and sampling design can strongly influence the ranges of measurements. Therefore, the contrast with Öztürk [50] could reflect both changing environmental pressure and methodological differences. Source strength and hydrodynamic confinement in harbour environments can also vary over time, complicating the attribution of obvious differences to long-term trends. Therefore, the present data are best interpreted as evidence of non-pristine conditions within the inner harbour that warrant continued attention rather than as a definitive temporal trend for the region.

Turkish comparisons further emphasise the site- and species-dependence. In İzmit Bay (Yalova), Cd and Pb in *P. caerulea* exceeded safety limits in some months, and associated THQ/TTHQ and carcinogenic risk values indicated a higher health concern than observed here [48]. In contrast, the gastropod *Rapana venosa* from the Eastern Black Sea showed THQ and TTHQ values below 1 [43]. These results indicate that exposure patterns and risk screening outcomes strongly depend on local sources, hydrography, and species ecology.

### 4.4. Trace Element-Related Non-Carcinogenic Health Risk Assessment

The non-carcinogenic risk screening provides one perspective on consumption-related safety for *P. caerulea* based on the analysed trace elements, but it does not address other seafood hazards outside the scope of this study. THQ and TTHQ were used, with values above 1 indicating potential concern [43]. All TTHQ values remained below 1, suggesting no exceedance of non-carcinogenic risk benchmarks under the high-consumption scenario. Similar outcomes have been reported in other studies on Black Sea seafood [31,43,53]. The margin to the benchmark was not uniform: the highest TTHQ occurred for 6-year-old children in autumn and approached the threshold, driven primarily by Cd. This alignment between risk estimates and seasonal tissue burdens indicates that single-season assessments may underestimate consumer exposure, which is seasonally dynamic.

To contextualise the measured concentrations within a food-safety framework, the Cd and Pb values were compared with the maximum levels specified for bivalve molluscs in European and Turkish contaminant legislation. In the European Union, these limits were formerly specified under Commission Regulation (EC) No 1881/2006 and are now included in the current Commission Regulation (EU) 2023/915 [54], which repealed and replaced Regulation (EC) No 1881/2006 [55]. In Türkiye, the corresponding limits are specified in the Turkish Food Codex Regulation on Contaminants [56]. These regulatory limits are not species-specific legal criteria for gastropods such as *P. caerulea*, but they provide a practical benchmark for interpreting the magnitude of Cd and Pb contamination. Using the moisture content applied in this study, the mean Cd and Pb concentrations corresponded to approximately 0.34 mg kg^−1^ wet weight and 0.03 mg kg^−1^ wet weight, respectively. These values were below the regulatory benchmark limits specified for bivalve molluscs in both European and Turkish legislation (Cd: 1.0 mg kg^−1^ ww; Pb: 1.5 mg kg^−1^ ww). Nevertheless, interpretation should remain cautious because taxon-specific differences in physiology, accumulation capacity, and consumption patterns mean that, in the absence of dedicated legal limits for limpets, bivalve-based thresholds cannot be directly transferred to *P. caerulea*.

### 4.5. Limitations

This study provides locally focused evidence on seasonal and fine-scale spatial variation in trace-element concentrations in *P. caerulea* from the Sinop inner harbour and a screening-level evaluation of associated non-carcinogenic risk. Some limitations should be considered in future studies.

• First, Spearman correlations describe covariation among trace elements in *P. caerulea* tissues; however, these relationships should be interpreted as descriptive rather than mechanistic because age was not determined and key environmental covariates (e.g., temperature, salinity, pH, and suspended particulate matter) were not measured concurrently, and they are not used here to infer causality or identify sources.
• Second, the spatial design was restricted to three closely spaced stations within a single inner-harbour system; therefore, the station effect reflects fine-scale within-harbour heterogeneity rather than broader coastal-scale variation. Accordingly, the stronger seasonal signal observed in this study should be interpreted within the investigated spatial scale.
• Third, the measured trace-element concentrations and THQ/TTHQ values represent a site-specific exposure-screening scenario for field-collected *P. caerulea* from this harbour and do not capture the full range of contamination that may occur across other harvesting areas or retail-market products. Furthermore, formal quantitative data on harvesting intensity at this specific site were not available, and market-purchased samples were beyond the scope of this study.

These considerations do not diminish the present dataset’s value for within-harbour biomonitoring and local exposure screening; rather, they define the conclusions’ spatial and interpretive scope. Future studies incorporating multiple harvesting locations, minimally impacted reference sites, open-coast stations, market-chain samples, concurrent environmental measurements, and multi-year observations would further strengthen the generalisability and mechanistic interpretation of the findings.

## 5. Conclusions

This study provides insights into trace element contamination and associated non-carcinogenic health risks in edible *P. caerulea* from the Sinop inner harbour. Seasonality was the primary bioaccumulation driver, with peak concentrations generally occurring in autumn. From the perspective of trace-element-related non-carcinogenic risk, TTHQ values remained below 1 under the assessed high-consumption scenario, although Cd contributed the most to cumulative risk and values approached the threshold for children in autumn. These findings highlight the importance of incorporating seasonal variability into locally focused trace-element exposure screening and support the use of *P. caerulea* as a biomonitor in semi-enclosed harbour environments. Future work should include minimally impacted reference sites and open-coast stations to extend inference beyond the harbour system.

## Figures and Tables

**Table 1:** Results of the permutational multivariate analysis of variance (PERMANOVA) testing the effects of season, station, and their interaction on multivariate concentrations of trace elements in the Mediterranean limpet (*Patella caerulea*). The test was performed sequentially (Type I) using a Bray–Curtis dissimilarity matrix with 999 permutations.

Source	Df	Sum of Sqs	R^2^	F	*p*
Season	3	0.0195	0.769	132.270	0.001
Station	2	0.0028	0.112	28.750	0.001
Season:Station	6	0.0018	0.072	6.230	0.001
Residual	24	0.0012	0.047		
Total	35	0.0253	1.000		

**Table 2:** Comparison of trace element concentrations in the Mediterranean limpet (*Patella caerulea*) from various marine regions. All concentrations are expressed in mg kg^−1^ dry weight (dw).

Trace Elements										Location	References
Cd	Cu	Fe	Ni	Pb	Zn	Mn	Co	Hg	As		
0.042–0.391	0.142–0.998	n.d.	0.111–1.944	0.265–2.625	0.128–0.770	n.d.	n.d.	n.d.	n.d.	Black Sea (Sinop), Türkiye	Öztürk [50]
3.30 ± 0.37– 6.30 ± 2.08	1.21 ± 0.30– 2.35 ± 0.67			0.14 ± 0.05– 1.52 ± 0.34	3.5 ± 1.6– 14.6 ± 3.8					Favignana Island, Sicily, Italy	Campanella et al. [22]
1.7–11.8	0.47–3.79			0.06–2.18	2.2–19.1					Favignana Island, Sicily, Italy	Cubadda et al. [24]
0.15 ± 0.06– 0.16 ± 0.05				0.18 ± 0.13– 0.28 ± 0.15						Ionian Sea, Italy	Storelli and Marcotrigiano [26]
0.24 ± 0.03– 0.68 ± 0.03	1.09 ± 0.01– 5.58 ± 0.18		0.39 ± 0.03– 1.60 ± 0.05	0.05 ± 0.0– 0.70 ± 0.03	3.70 ± 0.02– 13.7 ± 0.15					Iskenderun Gulf, Türkiye	Yüzereroğlu et al. [27]
0.78 ± 0.24– 1.63 ± 0.27	5.59 ± 0.47– 9.29 ± 1.43	1.86 ± 0.17– 2.59 ± 0.18	3.00 ± 1.12– 3.43 ± 0.8	3.51 ± 0.67– 3.61 ± 0.8						The Tunisian North Coast	Belkhodja and Romdhane [21]
0.605 ± 0.0252– 1.065 ± 0.0858				0.722 ± 0.0757– 1.848 ± 0.0711						Gulf of Gabes, Tunisia	Rabaoui et al. [25]
	7.2–95.8				38.1–126.0					Iranian Coasts	Bordbar et al. [51]
3.6	16.76	n.d.	n.d.	32.41	27.36	n.d.	n.d.	n.d.	n.d.	Mediterranean Sea	El-Adl and Bream [52]
0.004 ± 0.001– 0.065 ± 0.019	n.d.–0.249 ± 0.051	1.85 ± 0.89– 76.0 ± 5.7	0.021 ± 0.002– 0.339 ± 0.067	0.010 ± 0.003– 0.191 ± 0.028	0.26 ± 0.06– 1.66 ± 0.23					Aegean Sea, Türkiye	Aydın-Önen and Öztürk [20]
5.7	8.1	306.2	2.1	19.8	34	2.1	4.4	n.d.	n.d.	Mediterranean Sea	Duysak and Azdural [45]
0.104 ± 0.03– 2.318 ± 2.99	1.339 ± 0.42– 4.417 ± 1.15		0.850 ± 0.31– 4.544 ± 2.90	0.479 ± 0.14– 3.58 ± 0.94	12.61 ± 1.91– 29.77 ± 16.1					Pontine Islands, Italy	Conti et al. [23]
0.23 ± 0.21	0.014 ± 0.01– 0.06 ± 0.07	0.73 ± 0.31– 9.10 ± 4.72	0.11 ± 0.04– 0.60 ± 0.25	0.21 ± 0.11– 1.29 ± 0.36	1.54 ± 1.24– 8.21 ± 7.48					Tunisian Mediterranean Coast	Zaidi et al. [4]
2.21	2.45	1134	16.31	1.59	21.12	n.d.	n.d.	n.d.	n.d.	Sea of Marmara, Türkiye	Türk Çulha et al. [48]
1.667 ± 0.081	1.191 ± 0.052	152.102 ± 6.777	0.290 ± 0.011	0.135 ± 0.005	7.308 ± 0.430		0.031 ± 0.002	0.012 ± 0.001	1.473 ± 0.081	Black Sea (Sinop), Türkiye	This study *

* Mean ± 95% CI.

## Data Availability

The data supporting the findings of this study are available upon request from the corresponding author.

## Supplementary Materials

The following supporting information can be downloaded at: https://www.mdpi.com/article/10.3390/life16050806/s1, Table S1: Summary of Target Hazard Quotient (THQ) and Total Target Hazard Quotient (TTHQ) values calculated for children and adults based on Mediterranean limpet (*Patella caerulea*) tissue composites from the Sinop Peninsula, Black Sea, Türkiye.

## Abbreviations

The following abbreviations are used in this manuscript:

Abbreviation	Full term
AT	Averaging time
BW	Body weight
CI	Confidence interval
CRM	Certified reference material
dw	Dry weight
ED	Exposure duration
EF	Exposure frequency
EU	European Union
FIR	Food ingestion rate
ICP-MS	Inductively coupled plasma–mass spectrometry
PCA	Principal component analysis
PERMANOVA	Permutational multivariate analysis of variance
QA/QC	Quality assurance/quality control
RfD_o_	Oral reference dose
St.	Station
THQ	Target hazard quotient
TTHQ	Total target hazard quotient
USEPA	United States Environmental Protection Agency
ww	Wet weight
