# Supplementary material for: Seasonal Trace Element Contamination and Health Risk Assessment of the Mediterranean Limpet (Patella caerulea) from the Southern Black Sea
Source: Life (Basel). 2026 May 13;16(5):806. doi: 10.3390/life16050806 (PMC13208419; doi:10.3390/life16050806)
Supplement: Supplementary file 1 [file life-16-00806-s001.zip › life-4174501-supplementary.pdf]

Table S1. Summary of Target Hazard Quotient (THQ) and Total THQ (TTHQ) values (mean ± 95% CI) calculated for children (aged 6–12 years) and adults based on the 36 analytical determinations obtained from Mediterranean limpet (*Patella caerulea*) tissue composites collected from three sampling stations along the Sinop Peninsula, Black Sea, Türkiye.

	Winter			Spring			Summer			Autumn		
	St. 1	St. 2	St. 3	St. 1	St. 2	St. 3	St. 1	St. 2	St. 3	St. 1	St. 2	St. 3
Age 6 years/o												
Mn	0.0023 ± 0.0002	0.0027 ± 0.0001	0.0026 ± 0.0000	0.0024 ± 0.0001	0.0028 ± 0.0002	0.0027 ± 0.0002	0.0028 ± 0.0001	0.0033 ± 0.0002	0.0032 ± 0.0001	0.0029 ± 0.0000	0.0034 ± 0.0000	0.0033 ± 0.0001
Fe	0.0619 ± 0.0028	0.0613 ± 0.0017	0.0642 ± 0.0012	0.0657 ± 0.0021	0.0651 ± 0.0011	0.0681 ± 0.0030	0.0764 ± 0.0018	0.0748 ± 0.0011	0.0783 ± 0.0016	0.0856 ± 0.0037	0.0853 ± 0.0029	0.0900 ± 0.0045
Co	0.0260 ± 0.0026	0.0337 ± 0.0010	0.0318 ± 0.0007	0.0278 ± 0.0034	0.0352 ± 0.0010	0.0336 ± 0.0023	0.0331 ± 0.0015	0.0420 ± 0.0008	0.0404 ± 0.0013	0.0334 ± 0.0008	0.0431 ± 0.0027	0.0408 ± 0.0024
Ni	0.0041 ± 0.0004	0.0045 ± 0.0005	0.0043 ± 0.0002	0.0043 ± 0.0000	0.0048 ± 0.0004	0.0046 ± 0.0003	0.0050 ± 0.0002	0.0054 ± 0.0001	0.0053 ± 0.0003	0.0051 ± 0.0004	0.0057 ± 0.0006	0.0054 ± 0.0002
Cu	0.0081 ± 0.0003	0.0090 ± 0.0002	0.0085 ± 0.0000	0.0086 ± 0.0003	0.0096 ± 0.0006	0.0091 ± 0.0007	0.0104 ± 0.0002	0.0115 ± 0.0003	0.0109 ± 0.0007	0.0108 ± 0.0006	0.0120 ± 0.0003	0.0114 ± 0.0005
Zn	0.0081 ± 0.0002	0.0061 ± 0.0004	0.0069 ± 0.0001	0.0086 ± 0.0004	0.0065 ± 0.0005	0.0075 ± 0.0001	0.0098 ± 0.0010	0.0073 ± 0.0000	0.0084 ± 0.0001	0.0112 ± 0.0016	0.0083 ± 0.0003	0.0095 ± 0.0003
Cd	0.4522 ± 0.0401	0.5046 ± 0.0143	0.4625 ± 0.0569	0.4815 ± 0.0755	0.5369 ± 0.0072	0.4926 ± 0.0218	0.5871 ± 0.0225	0.6469 ± 0.0121	0.6008 ± 0.0037	0.6253 ± 0.0045	0.6954 ± 0.0325	0.6404 ± 0.0140
Pb	0.0202 ± 0.0003	0.0214 ± 0.0005	0.0191 ± 0.0008	0.0214 ± 0.0004	0.0227 ± 0.0018	0.0202 ± 0.0005	0.0240 ± 0.0007	0.0252 ± 0.0013	0.0226 ± 0.0014	0.0251 ± 0.0007	0.0263 ± 0.0012	0.0237 ± 0.0014
Hg	0.0389 ± 0.0025	0.0310 ± 0.0022	0.0350 ± 0.0013	0.0410 ± 0.0005	0.0335 ± 0.0023	0.0374 ± 0.0027	0.0507 ± 0.0019	0.0405 ± 0.0016	0.0453 ± 0.0016	0.0516 ± 0.0013	0.0419 ± 0.0016	0.0469 ± 0.0028
As	0.0141 ± 0.0001	0.0146 ± 0.0003	0.0142 ± 0.0002	0.0149 ± 0.0008	0.0154 ± 0.0013	0.0151 ± 0.0006	0.0165 ± 0.0002	0.0169 ± 0.0009	0.0167 ± 0.0007	0.0173 ± 0.0002	0.0178 ± 0.0006	0.0246 ± 0.0004
**TTHQ**	**0.6359 ± 0.0412**	**0.6890 ± 0.0155**	**0.6493 ± 0.0586**	**0.6762 ± 0.0733**	**0.7325 ± 0.0088**	**0.6908 ± 0.0223**	**0.8160 ± 0.0219**	**0.8739 ± 0.0116**	**0.8320 ± 0.0039**	**0.8683 ± 0.0042**	**0.9392 ± 0.0342**	**0.8959 ± 0.0066**
**Age 12 years/o**												
Mn	0.0011 ± 0.0001	0.0013 ± 0.0001	0.0013 ± 0.0000	0.0012 ± 0.0000	0.0014 ± 0.0001	0.0014 ± 0.0001	0.0014 ± 0.0000	0.0017 ± 0.0001	0.0016 ± 0.0000	0.0014 ± 0.0000	0.0017 ± 0.0000	0.0016 ± 0.0001
Fe	0.0309 ± 0.0014	0.0307 ± 0.0008	0.0321 ± 0.0006	0.0329 ± 0.0011	0.0325 ± 0.0005	0.0341 ± 0.0015	0.0382 ± 0.0009	0.0374 ± 0.0006	0.0392 ± 0.0008	0.0428 ± 0.0019	0.0426 ± 0.0015	0.0450 ± 0.0022
Co	0.0130 ± 0.0013	0.0169 ± 0.0005	0.0159 ± 0.0004	0.0139 ± 0.0017	0.0176 ± 0.0005	0.0168 ± 0.0012	0.0166 ± 0.0008	0.0210 ± 0.0004	0.0202 ± 0.0006	0.0167 ± 0.0004	0.0215 ± 0.0014	0.0204 ± 0.0012
Ni	0.0020 ± 0.0002	0.0023 ± 0.0003	0.0022 ± 0.0001	0.0022 ± 0.0000	0.0024 ± 0.0002	0.0023 ± 0.0002	0.0025 ± 0.0001	0.0027 ± 0.0001	0.0026 ± 0.0001	0.0026 ± 0.0002	0.0028 ± 0.0003	0.0027 ± 0.0001
Cu	0.0041 ± 0.0002	0.0045 ± 0.0001	0.0043 ± 0.0000	0.0043 ± 0.0001	0.0048 ± 0.0003	0.0045 ± 0.0003	0.0052 ± 0.0001	0.0057 ± 0.0001	0.0055 ± 0.0004	0.0054 ± 0.0003	0.0060 ± 0.0002	0.0057 ± 0.0002
Zn	0.0041 ± 0.0001	0.0031 ± 0.0002	0.0035 ± 0.0001	0.0043 ± 0.0002	0.0033 ± 0.0002	0.0038 ± 0.0000	0.0049 ± 0.0005	0.0037 ± 0.0000	0.0042 ± 0.0000	0.0056 ± 0.0008	0.0041 ± 0.0002	0.0048 ± 0.0001
Cd	0.2261 ± 0.0201	0.2523 ± 0.0071	0.2313 ± 0.0285	0.2407 ± 0.0377	0.2684 ± 0.0036	0.2463 ± 0.0109	0.2936 ± 0.0112	0.3235 ± 0.0061	0.3004 ± 0.0018	0.3127 ± 0.0023	0.3477 ± 0.0163	0.3202 ± 0.0070
Pb	0.0101 ± 0.0001	0.0107 ± 0.0003	0.0095 ± 0.0004	0.0107 ± 0.0002	0.0113 ± 0.0009	0.0101 ± 0.0003	0.0120 ± 0.0003	0.0126 ± 0.0006	0.0113 ± 0.0007	0.0126 ± 0.0003	0.0132 ± 0.0006	0.0118 ± 0.0007
Hg	0.0195 ± 0.0012	0.0155 ± 0.0011	0.0175 ± 0.0006	0.0205 ± 0.0002	0.0168 ± 0.0012	0.0187 ± 0.0013	0.0254 ± 0.0010	0.0203 ± 0.0008	0.0226 ± 0.0008	0.0258 ± 0.0007	0.0209 ± 0.0008	0.0234 ± 0.0014
As	0.0070 ± 0.0000	0.0073 ± 0.0002	0.0071 ± 0.0001	0.0074 ± 0.0004	0.0077 ± 0.0006	0.0075 ± 0.0003	0.0083 ± 0.0001	0.0085 ± 0.0004	0.0084 ± 0.0003	0.0087 ± 0.0001	0.0089 ± 0.0003	0.0123 ± 0.0002
**TTHQ**	**0.3179 ± 0.0206**	**0.3445 ± 0.0078**	**0.3246 ± 0.0293**	**0.3381 ± 0.0366**	**0.3662 ± 0.0044**	**0.3454 ± 0.0112**	**0.4080 ± 0.0110**	**0.4370 ± 0.0058**	**0.4160 ± 0.0019**	**0.4341 ± 0.0021**	**0.4696 ± 0.0171**	**0.4479 ± 0.0033**
**Adult**												
Mn	0.0007 ± 0.0001	0.0008 ± 0.0000	0.0008 ± 0.0000	0.0007 ± 0.0000	0.0009 ± 0.0001	0.0008 ± 0.0001	0.0009 ± 0.0000	0.0010 ± 0.0001	0.0010 ± 0.0000	0.0009 ± 0.0000	0.0010 ± 0.0000	0.0010 ± 0.0000
Fe	0.0189 ± 0.0009	0.0187 ± 0.0005	0.0196 ± 0.0004	0.0201 ± 0.0006	0.0199 ± 0.0003	0.0208 ± 0.0009	0.0233 ± 0.0005	0.0229 ± 0.0003	0.0239 ± 0.0005	0.0261 ± 0.0011	0.0261 ± 0.0009	0.0275 ± 0.0014
Co	0.0080 ± 0.0008	0.0103 ± 0.0003	0.0097 ± 0.0002	0.0085 ± 0.0011	0.0107 ± 0.0003	0.0103 ± 0.0007	0.0101 ± 0.0005	0.0128 ± 0.0002	0.0123 ± 0.0004	0.0102 ± 0.0002	0.0132 ± 0.0008	0.0125 ± 0.0007
Ni	0.0012 ± 0.0001	0.0014 ± 0.0002	0.0013 ± 0.0001	0.0013 ± 0.0000	0.0015 ± 0.0001	0.0014 ± 0.0001	0.0015 ± 0.0001	0.0017 ± 0.0000	0.0016 ± 0.0001	0.0016 ± 0.0001	0.0017 ± 0.0002	0.0017 ± 0.0001
Cu	0.0025 ± 0.0001	0.0028 ± 0.0001	0.0026 ± 0.0000	0.0026 ± 0.0001	0.0029 ± 0.0002	0.0028 ± 0.0002	0.0032 ± 0.0001	0.0035 ± 0.0001	0.0033 ± 0.0002	0.0033 ± 0.0002	0.0037 ± 0.0001	0.0035 ± 0.0002
Zn	0.0025 ± 0.0001	0.0019 ± 0.0001	0.0021 ± 0.0000	0.0026 ± 0.0001	0.0020 ± 0.0001	0.0023 ± 0.0000	0.0030 ± 0.0003	0.0022 ± 0.0000	0.0026 ± 0.0000	0.0034 ± 0.0005	0.0025 ± 0.0001	0.0029 ± 0.0001
Cd	0.1382 ± 0.0123	0.1542 ± 0.0044	0.1413 ± 0.0174	0.1471 ± 0.0231	0.1640 ± 0.0022	0.1505 ± 0.0067	0.1794 ± 0.0069	0.1977 ± 0.0037	0.1836 ± 0.0011	0.1911 ± 0.0014	0.2125 ± 0.0099	0.1957 ± 0.0043
Pb	0.0062 ± 0.0001	0.0065 ± 0.0002	0.0058 ± 0.0002	0.0065 ± 0.0001	0.0069 ± 0.0006	0.0062 ± 0.0002	0.0073 ± 0.0002	0.0077 ± 0.0004	0.0069 ± 0.0004	0.0077 ± 0.0002	0.0080 ± 0.0004	0.0072 ± 0.0004
Hg	0.0119 ± 0.0008	0.0095 ± 0.0007	0.0107 ± 0.0004	0.0125 ± 0.0001	0.0102 ± 0.0007	0.0114 ± 0.0008	0.0155 ± 0.0006	0.0124 ± 0.0005	0.0138 ± 0.0005	0.0158 ± 0.0004	0.0128 ± 0.0005	0.0143 ± 0.0008
As	0.0043 ± 0.0000	0.0045 ± 0.0001	0.0043 ± 0.0001	0.0045 ± 0.0002	0.0047 ± 0.0004	0.0046 ± 0.0002	0.0051 ± 0.0001	0.0052 ± 0.0003	0.0051 ± 0.0002	0.0053 ± 0.0001	0.0054 ± 0.0002	0.0075 ± 0.0001
**TTHQ**	**0.1943 ± 0.0126**	**0.2105 ± 0.0047**	**0.1984 ± 0.0179**	**0.2066 ± 0.0224**	**0.2238 ± 0.0027**	**0.2111 ± 0.0068**	**0.2493 ± 0.0067**	**0.2670 ± 0.0035**	**0.2542 ± 0.0012**	**0.2653 ± 0.0013**	**0.2870 ± 0.0105**	**0.2737 ± 0.0020**
